# GenomicInteractions: An R/Bioconductor package for manipulating and investigating chromatin interaction data

**DOI:** 10.1186/s12864-015-2140-x

**Published:** 2015-11-17

**Authors:** Nathan Harmston, Elizabeth Ing-Simmons, Malcolm Perry, Anja Barešić, Boris Lenhard

**Affiliations:** Computational Regulatory Genomics, MRC Clinical Sciences Centre, Faculty of Medicine, Imperial College, London, W12 0NN UK; Program in Cardiovascular and Metabolic Disease, Duke-NUS Graduate Medical School, 8 College Road, Singapore, 169857 Singapore

## Abstract

**Background:**

Precise quantitative and spatiotemporal control of gene expression is necessary to ensure proper cellular differentiation and the maintenance of homeostasis. The relationship between gene expression and the spatial organisation of chromatin is highly complex, interdependent and not completely understood. The development of experimental techniques to interrogate both the higher-order structure of chromatin and the interactions between regulatory elements has recently lead to important insights on how gene expression is controlled. The ability to gain these and future insights is critically dependent on computational tools for the analysis and visualisation of data produced by these techniques.

**Results and conclusion:**

We have developed *GenomicInteractions*, a freely available R/Bioconductor package designed for processing, analysis and visualisation of data generated from various types of chromosome conformation capture experiments. The package allows the easy annotation and summarisation of large genome-wide datasets at both the level of individual interactions and sets of genomic features, and provides several different methods for interrogating and visualising this type of data. We demonstrate this package’s utility by showing example analyses performed on interaction datasets generated using Hi-C and ChIA-PET.

**Electronic supplementary material:**

The online version of this article (doi:10.1186/s12864-015-2140-x) contains supplementary material, which is available to authorized users.

## Background

Metazoan gene expression is controlled through the complex interplay of transcription factors, histone modifications and regulatory elements [1, 2] in three-dimensional nuclear space [3]. Gene expression is typically regulated by both the gene’s core and proximal promoters and through the action of distal elements such as enhancers [4] and insulators [5]. Physical interactions between these elements and their cognate promoters are currently thought to be a major mechanism for quantitatively and spatiotemporally regulating gene expression [6]. The positioning of chromosomes in the nucleus [7–10] and the organisation of chromatin at multiple scales [11, 12] have important roles in controlling the dynamics and specificity of these interactions, although the mechanisms involved are not completely understood. Information on how the spatial organisation of chromosomes impacts the regulation of gene expression is becoming increasingly available due to the development of experimental techniques to interrogate this phenomenon in a genome-wide manner [13].

Chromosome conformation capture methods have been developed for investigating chromatin interactions at both the level of individual loci (i.e. 3C [14], 4C [15], 5C [16], T2C [17]) and genome-wide (i.e. Capture-C [18], Hi-C [19, 20], ChIA-PET [21]). These methods work by cross-linking regions of genomic DNA that are in close physical proximity and thereby allowing the identification of interactions between genomic loci by the capture and sequencing of these regions. ChIA-PET (Chromatin Interaction Analysis with Paired-End Tag sequencing) allows for the investigation of interactions that are mediated by or associated with a specific protein (e.g. PolII [21]) or histone modification (e.g. H3K4me2 [22]), which is accomplished by performing a chromatin-immunoprecipitation step after crosslinking. The resulting data can then be used to generate interaction maps or networks detailing chromatin interactions, focusing either on specific genes and elements or genome-wide.

These methods have provided insights into the 3D organisation of chromatin across multiple cell types and conditions. Most interactions between genomic regions occur within the same chromosome (*cis*-interactions), with only a small number of interactions occurring reproducibly between elements on different chromosomes (*trans*-interactions) [23]. Chromatin is organised into distinct topologically associated domains (TADs) [12], with regulatory elements and genes preferentially interacting within the same TAD, and at the larger scale TADs are organised into compartments of active/inactive chromatin [19]. Both genes and enhancers are promiscuous with respect to their interaction partners, with genes able to interact with multiple enhancers and, less frequently, enhancers able to regulate multiple promoters [24]. The interaction landscape of a promoter is often highly dynamic and cell-type specific [25], with changes in its interaction partners thought to play a role in regulating its expression during development and differentiation [26, 27]. These findings were made possible not only by advances in experimental techniques but also because of the development of statistical and computational methods for data processing, filtering, normalisation and visualisation [19, 28–33], and currently there is considerable work on developing new statistical methodologies for analysing this type of data [34, 35].

Here, we present *GenomicInteractions*, an R/Bioconductor [36] package for the manipulation, annotation and visualisation of various types of chromatin interaction data, e.g. Hi-C, ChIA-PET. The development of this software was motivated by the lack of a general platform to analyse and visualise chromatin interaction data. Existing analysis tools are mostly standalone packages (e.g. *HOMER*, *ChIA-PET tool*), which do not have interfaces to the popular R/Bioconductor tools for genomic data analysis. Current R/Bioconductor packages for chromatin interaction data are generally specialised for a specific data type (e.g. HiC: *diffHiC* [37], *HiTC* [38], *GOTHiC* [39]; 4C: *r3Cseq* [32], *Basic4CSeq* [40], *FourCSeq* [41]). Most of these packages take BAM files as input and provide data processing and normalisation and visualisation functions. In contrast, *GenomicInteractions* can be used with any type of chromatin interaction data in a range of formats, and is designed for interactive data exploration and visualisation. The ability to import data from several formats and its integration with existing Bioconductor packages facilitates the integrative analysis of data from different experiments, for example combining ChIP-seq signal or gene expression data with interaction data. We describe the main features of this package and demonstrate its utility and novel features by analysing two different chromatin interaction datasets.

## Implementation

*GenomicInteractions* is a publicly available Bioconductor package for the handling of chromatin interaction data. It follows the same naming conventions as core Bioconductor packages, such as *GenomicRanges* [42]. We provide vignettes detailing the use of *GenomicInteractions* in analysing both Hi-C and ChIA-PET data.

### Interoperability and integration with other Bioconductor packages

**Fig. 1:**
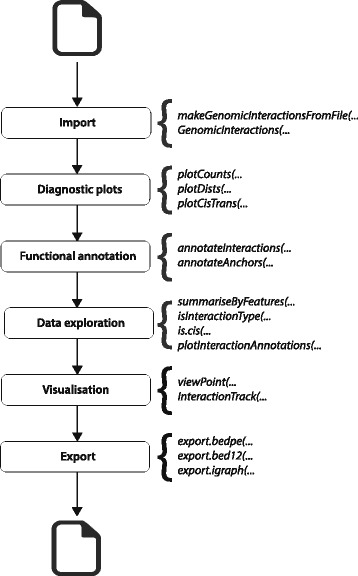
Typical workflow for analysing a chromatin interaction dataset. A workflow may involve investigating which distal regions a gene of interest is interacting with, or may involve summarising the number and types of interactions a set of regions is involved in. In order to accomplish this, the relevant data needs to be imported into R, filtered appropriately, annotated with information on genes and/or regions and interrogated. During this process, a researcher can visualise the imported data, focusing either on genome-wide or locus-specific features. Finally, the data can be exported for use by other software packages. Methods defined in *GenomicInteractions* that can be used to perform each task are shown in *italics*

Our package is designed to be as high-level as possible in order to allow its use in a wide range of analyses using different types of chromatin interaction data. Although the methods used to generate and process chromatin interaction data vary, the conceptual structure of the data is a series of pairs of genomic regions involved in the interactions (known as anchors) and data associated with each pair of regions e.g. supporting counts, p-value and false discovery rate (FDR). We define an S4 class, which encapsulates this structure and allows the easy manipulation and investigation of interactions stored within it. Anchor regions are stored as *GenomicRanges* objects, allowing individual anchors to be efficiently queried and annotated with relevant data and metadata. As with any analysis of biological data, the specific steps involved depend on the experimental design and on the biological questions being asked. However, most tasks can be grouped together and organised into a workflow structure (Fig. 1), regardless of how the data was generated originally.

### Data import

The package can import chromatin interaction data stored in several formats, including the output from common processing tools [43], e.g. *HOMER* [28], *ChIA-PET tool* [30], and from standard formats, e.g. bed12, bedpe and BAM. This allows users to easily import data processed using existing tools, while also providing methods for directly manipulating aligned reads (e.g. merging interactions between predefined anchors, removing positional duplicates and determining thresholds for self-ligation events).

### Determining self-ligation thresholds

The package contains implementations of two methods for calculating thresholds to separate reads into those that are the result from self-ligations versus those that arise from inter-ligations. This threshold can be identified by comparing the distribution of paired-end reads mapping to the same-strand against those aligning to different strands. The paired-end reads are binned by distance and the ratio is calculated for each bin. A binomial test is available for testing whether this ratio is different from the expected 50:50 ratio in a specific bin. Additionally, we provide an implementation of the method described in Heidari et al. [44, 45], where the cut-off is determined by examining the strand distribution of reads which span over long distances.

### Interaction summaries

**Fig. 2:**
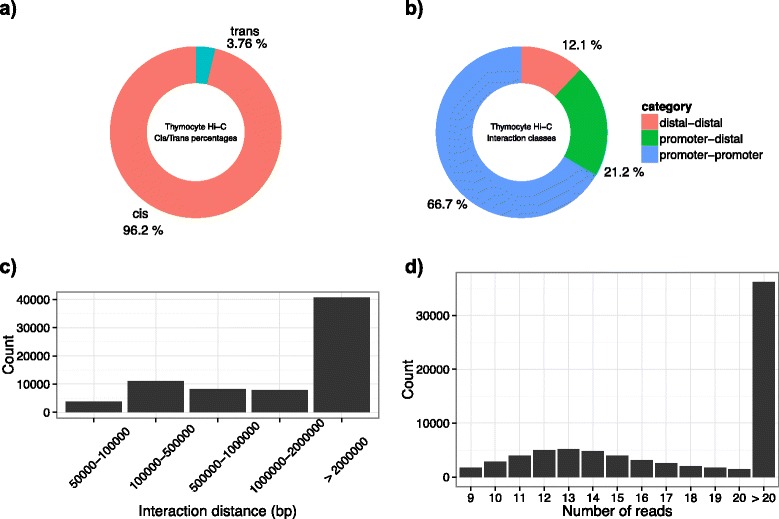
Summary statistics of mouse double positive (CD4+ CD8+) thymocyte Hi-C data generated using the *plotSummaryStats* function from *GenomicInteractions*. **a** Donut plot showing percentage of *cis*/*trans* interactions within the dataset. **b** Donut plot describing the distribution of types of interaction observed. **c** Distribution of interaction distances between anchor regions (base pairs) **d** Number of reads supporting each interaction

**Fig. 3:**
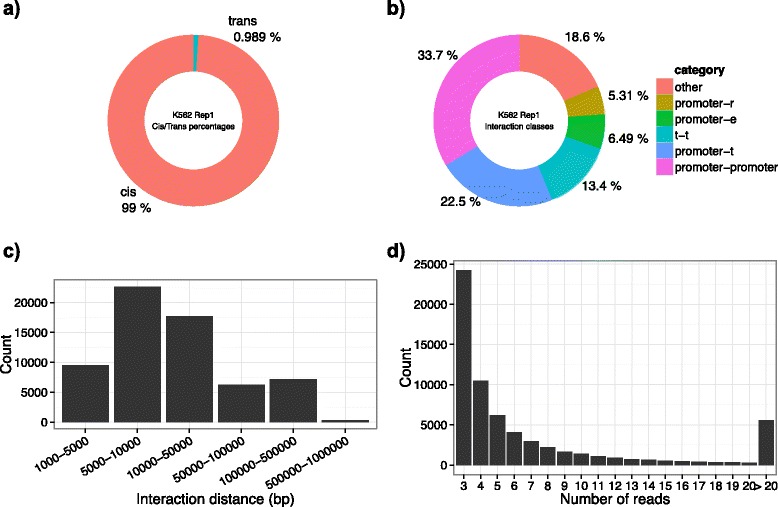
Summary statistics of K562 RNAPII ChIA-PET dataset (Replicate 1) generated using the *plotSummaryStats* function from *GenomicInteractions*. K562 ChIA-PET data for PolII (8WG16) was taken from Li et al. [21] and filtered as described in the associated text. **a** Number of *cis*/*trans* interactions **b** Donut plot of number of interaction classes (promoter—2.5 kb around an Ensembl gene TSS, r—repressed region, e—enhancer or weak enhancer, t—transcribed region) **c** Distribution of interaction distances between anchor regions. **d** Number of reads supporting each interaction

We provide methods for creating various diagnostic plots (see Figs. 2 and 3), including visualising the distribution of distances spanned by the interactions, the proportion of *cis-* and *trans*-interactions in the dataset, and the number of reads supporting each interaction.

### Annotation, interrogation of interacting regions

The package allows both interactions and genomic features/regions of interest to be annotated and examined easily. Each anchor region can be annotated with whether or not it overlaps a region of interest (which specifies the class of the anchor e.g. promoter) and an identifier specifying that region (e.g. a gene identifier). For example, this allows anchors to be annotated with which gene promoters, transcription factor binding peaks or chromatin states they overlap with. This in turn allows the extraction of all interactions that are between pairs of promoters (promoter:promoter interactions), or between other features of interest (e.g. promoter:enhancer or enhancer:enhancer interactions). A *GenomicInteractions* object can be queried and filtered based on user-defined criteria: for example, it is straightforward to subset the object to only contain interactions within or between specific chromosomes or specific features. Users can summarise interactions at the level of individual genomic features, identifying the total number of interactions a feature is involved in, or the number of other features with which it interacts. This makes it possible to identify gene promoters involved in many interactions with distal/enhancer regions, thus resolving promoter:enhancer interactions at complex loci with non-linear arrangement of genes and the regulatory elements that control them [27, 45].

### Visualisation of interactions

**Fig. 4:**
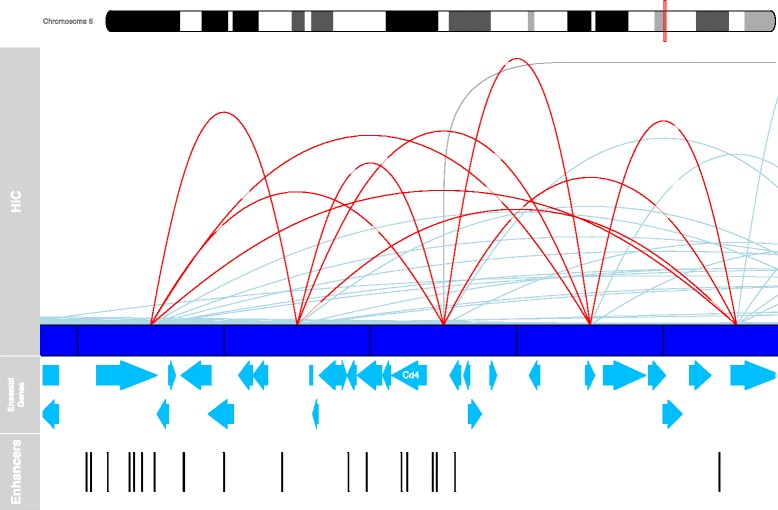
The interaction landscape spanning 500 kb around the promoter of *Cd4* in mouse (mm9) double positive (CD4+ CD8+) thymocytes. The height of each curve corresponds to the number of PETs supporting that interaction. The resolution of the data is not high enough to detect interactions within the *Cd4* gene region, however numerous interactions with both neighbouring 100 kb regions and distal regions on the same chromosome are observed (shown in light blue). The 100 kb region containing *Cd4* also participates in at least one *trans*-chromosomal interaction (dark grey line). Tracks displaying Ensembl protein-coding genes and enhancers active in the mouse thymus [52] present in the region are also shown

**Fig. 5:**
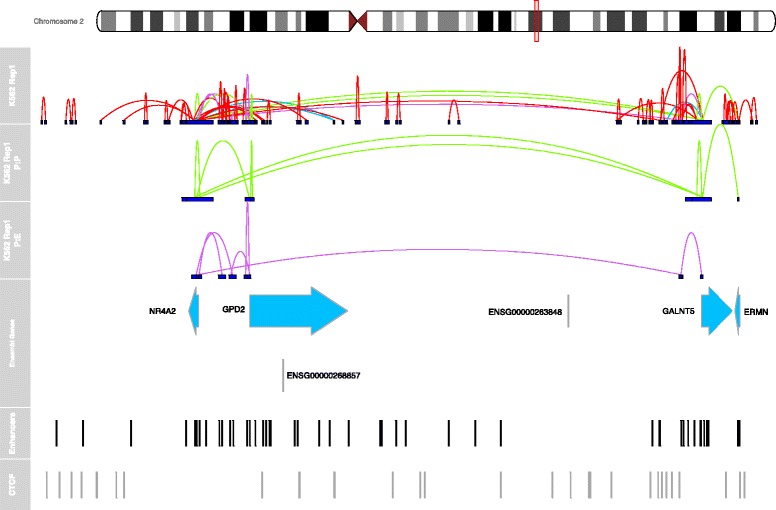
The interaction landscape of *NR4A2* in K562 cells (hg19) as determined by ChIA-PET (chr2:156898860–158248860). See associated text for more details on processing. All identified interactions are shown in the top panel, with promoter:promoter interactions and promoter:enhancer interactions shown in the panels below. This gene is involved in interactions with the promoters of two nearby genes (*GPD2* and *GALNT5*) and a small number of enhancers. The height of each curve corresponds to negative logarithm of the FDR for each interaction. Promoter:promoter interactions are shown in green, promoter-enhancer interactions in purple, promoter:distal interactions in orange and promoter:ctcf interactions are displayed in blue

The proportion of interactions between different classes of features can be calculated and visualised (Figs. 2 and 3). It is also possible to generate a *virtual 4C* viewpoint-style plot of all interactions involving a region(s) of interest, e.g. a specific promoter, or around a set of transcription factor binding sites. In addition, the package provides methods for visualising interactions and features within a defined genomic region by representing interactions between anchors as curves (Figs. 4 and 5) via integration with the Gviz visualisation library [46].

### Data export

Finally, users can export their dataset to a variety of output formats for further analysis with other tools. We have provided methods for exporting a *GenomicInteractions* object to bed12 format, which can be used, for example, to visualise the interactions in the UCSC Genome Browser [47]. It is also possible to convert the interaction data into a graph format compatible with the igraph library [48], allowing the examination of data using network analysis approaches.

## Results

### Usage examples

#### Investigating Hi-C data from mouse thymocytes

Here, we describe using *GenomicInteractions* to perform an example analysis of Hi-C data generated using mouse double positive (CD4+ CD8+) thymocytes [49] (GEO dataset GSE48763). All code and data required to reproduce this analysis can be found in Additional file 1. Two biological replicates, totalling about 203 M paired-end reads were aligned using *bowtie* [50]. Uniquely mappable reads were then pooled and processed using the *HOMER* software pipeline, to remove sources of noise and bias. This resulted in the identification of a set of 100 kb regions involved in significant interactions, taking both genomic distance and sequencing depth into account. *GenomicInteractions* has a built-in function to import data from the *HOMER* interaction file format.

This gives 74443 interactions at an FDR of 5 %. Almost all (96.2 %) of these are *cis*-chromosomal interactions, although many are long-range interactions across distances of more than 2 Mb. These properties can be quickly summarised using plotting functions provided in the package (Fig. 2a,c). Annotation of these interactions (as either promoters or distal elements) reveals that the majority are annotated as promoter:promoter interactions (Fig. 2b). This is partly due to the resolution of the Hi-C data; as the anchors are 100 kb, the chance that they will contain at least one promoter is high.

Figure 4 shows the interaction landscape around the 100 kb anchor that contains the promoter of the *Cd4* gene. CD4 is a cell surface protein that is a key cell identity marker for CD4+ CD8+ thymocytes. Its gene is highly expressed in these cells and is regulated by an intronic enhancer and multiple distal elements [51, 52]. Although the resolution of the data is not high enough to detect interactions within the *Cd4* gene region, numerous interactions with both neighbouring 100 kb regions and distal regions on the same chromosome are apparent. The 100 kb region containing *Cd4* also participates in at least one *trans*-chromosomal interaction (*grey line*, Fig. 4). These interactions could be investigated further using other chromosome conformation capture methods or DNA FISH.

#### Investigating ChIA-PET data from human K562 cells

K562 ChIA-PET data for PolII (8WG16) was taken from Li *et al.* [21] replicate 1 (GEO dataset GSE33664). This dataset has been processed using the ChIA-PET tool, with interactions supported by more than two PET counts and having an FDR < 0.05 considered as significant. All code and data required to replicate this analysis can be found in Additional file 2.

All interactions involving chrM were filtered from the dataset, resulting in 64554 unique interactions supported by 879351 PETs. The vast majority of interactions in this dataset occur in *cis*, with only 1 % (637) occurring *trans-*chromosomally (Fig. 3a). There are 166 interactions which span more than 1 Mb, some of which show interactions between regions over 17 Mb apart. These *super-long range* interactions were removed from further analysis. Only a small number (N = 508) of remaining interactions appear to span distances longer than 500 kb (Fig. 3c).

In order to more accurately define the promoter region of a gene, the robust DPI promoter set generated from the FANTOM5 data [53] was used to propose the TSS of each gene. Only genes coding for proteins, long intergenic non-coding RNAs (lincRNAs) or microRNAs (miRNAs) were considered. Promoter regions were defined as +/− 2.5 kb around this set of TSSs. Chromatin state annotations for K562 were obtained from Hoffman et al. [54]. *GenomicInteractions* relies on a user-defined order of importance of features in order to assign classes to individual anchors. Features were ordered as *promoter, t (*transcribed region*)* and *e* (enhancer or weak enhancer), *ctcf* (CTCF region) and *r* (repressed region). If an anchor lies within a region not covered by one of these annotations it was labelled as distal. The majority of interactions in this dataset appear to be between promoters and other promoters (N = 21694), with a large number of promoter:enhancer interactions (N = 4177) (see Fig. 3b). As expected [23], a number of enhancer:enhancer interactions were also observed (N = 1209).

Interaction data was summarised at the level of promoters, i.e. PET counts of all anchors overlapping the promoter regions of each gene have been summed together, which revealed the genes involved in the highest number of interactions genome-wide. 13215 of the 19358 genes examined were involved in some form of interaction as identified by ChIA-PET. The top ten genes ranked by total number of promoter:enhancer interactions are shown in Table 1. Some of these genes have been previously found to play important roles in haematopoiesis and leukaemia pathogenesis, e.g. *PIM1* [55]*, BCOR* [56]*, TNFRSF8* [57] and *NR4A2* [58]. The number of promoters and enhancers that interact with each promoter was also calculated. In some cases, due to the close genomic proximity of some enhancers and promoters it was not possible to distinguish which individual enhancer or promoter an interaction was involved with.

**Table 1:** Genes with the highest number of promoter:enhancer interactions in RNA Polymerase II ChIA-PET with 8WG16 antibody in human K562 cell line, replicate 1 [21], see associated text for more details on processing of this dataset

Ensembl ID	HGNC Symbol	Number of interactions	Number of promoter:promoter interactions	Number of promoters	Number of promoter:enhancer interactions	Number of enhancers
ENSG00000177000	*MTHFR*	934	686	7	233	9
ENSG00000011021	*CLCN6*	901	686	7	208	9
ENSG00000183337	*BCOR*	2585	2444	1	110	14
ENSG00000137193	*PIM1*	167	60	1	107	42
ENSG00000007944	*MYLIP*	721	485	2	97	38
ENSG00000120949	*TNFRSF8*	198	91	1	84	3
ENSG00000153234	*NR4A2*	671	541	2	82	5
ENSG00000253276	*CCDC71L*	303	186	3	76	12
ENSG00000116717	*GADD45A*	537	317	1	73	12
ENSG00000172216	*CEBPB*	414	265	8	65	21

A window size of 2.5 kb around TSSs, defined using the FANTOM5 robust tag cluster set, was used to define promoters. Enhancers and other chromatin states were defined using the Genome Segments K562 track. The number of interactions is calculated as the sum of the PET counts of all anchors overlapping the promoter region of a gene

*NR4A2* (also known as Nurr1) is a member of the steroid orphan nuclear receptor transcription factor superfamily. It is essential in neurogenesis and the maintenance of dopaminergic neurons [59], plays a role in the activation of *FOXP3* in regulatory T cells and in their differentiation and function [60] and has been associated with various types of cancer [61]. The interaction landscape of *NR4A2* is shown in Fig. 5. The promoter of NR4A2 is involved in interactions with the promoter of its neighbouring gene *GPD2* (located 93 kb away) and a promoter of the gene *GALNT5* (located 910 kb away). It is interacting with five putative enhancers, four of which are located within 100 kb of the promoter of NR4A2, with one located almost 900 kb away. This enhancer also has interactions with the promoter of *GALNT5* and appears to be bound by a number of factors in K562 including GATA2, PML, TAL1 and BCL3, all of which have been implicated in the leukemia or other forms of cancer [62–64].

## Conclusions

*GenomicInteractions* provides a set of tools to import, manipulate, visualise and mine chromatin interaction data in R. The package has the potential to serve as a starting point for different types of analyses, providing the ability to ask relevant questions about the chromatin interactome using data generated from a variety of experimental techniques. In this paper, we have shown how *GenomicInteractions* allows an end-user to reproducibly and efficiently perform analyses of two publicly available genome-wide chromatin interaction datasets. This allowed the identification and visualisation of regulatory elements that are interacting with a number of interesting genes, the identification of genes with the highest number of interactions and the characterisation of sets of those interactions. The package is available under a GPL-3 licence, and users and developers can easily extend the implemented functionality to match their specific analysis needs. In the future we are looking to extend this package with additional methods for normalising and processing the data, and expand the number of formats from which interaction data can be imported.

## Availability and requirements

*GenomicInteractions* is a publicly available Bioconductor package available from http://bioconductor.org/packages/GenomicInteractions/. Documentation is available on the Bioconductor website, and we provide vignettes describing two example analyses using publicly available ChIA-PET and Hi-C data. We also maintain a public github repository (https://github.com/ComputationalRegulatoryGenomicsICL/GenomicInteractions), and invite the community to submit or request additional functionality to incorporate into this package. This package requires R > = 3.0.1 and depends on several R/Bioconductor packages including Rsamtools, GenomicRanges, data.table, stringr, rtracklayer, ggplot2, gridExtra, igraph and Gviz.

All of the analyses and figures presented in the paper can be reproduced via the RMarkdown documents provided in the supplemental material using *GenomicInteractions* (version 1.3.6 available on Github*)*, which is available (as version 1.4.0) in Bioconductor 3.2.

## Additional files

Additional file 1:
**R script used to generate figures, tables and numbers used in the described analysis of Hi-C data.** (RMD 7 kb) Additional file 2:
**R script used to generate figures, tables and numbers used in the described analysis of ChIA-PET data.** (RMD 14 kb)

## Abbreviations

ChIA-PET: Chromatin interaction analysis with paired-end tag sequencing
TAD: Topologically associated domain
FANTOM: Functional annotation of the mammalian genome
PET: Paired-end tag
GEO: Gene expression omnibus
FDR: False discovery rate
T2C: Targeted chromatin capture
FISH: Fluorescence in situ hybridisation
DPI: Decomposition-based peak identification
